# SQUIRE arrives—with a plan for its own improvement

**DOI:** 10.1136/qshc.2008.030247

**Published:** 2008-09-26

**Authors:** David P Stevens, Richard Thomson

**Affiliations:** Quality Literature Program, Dartmouth Institute for Health Policy and Clinical Care, Lebanon, New Hampshire, USA

The increased focus on the epistemology—the theory of knowledge—that underlies both healthcare improvement and patient safety has brought a fresh vitality to the scholarly healthcare improvement literature.^1^ Into this discussion of epistemology come the newly revised guidelines for publication of healthcare improvement reports, SQUIRE (Standards for QUality Improvement Reporting Excellence).^2^

SQUIRE was originally promulgated by Davidoff and Batalden in *Quality and Safety in Health Care* in 2005 as draft guidelines to advance the scholarship of improvement and to address the underlying theory of experiential learning that is central to much of healthcare improvement research.^3^ Subsequently, with financial support from the Robert Wood Johnson Foundation, a consensus conference of editors and improvement scholars was conducted in Boston in April 2007, and the original draft document was revised following the conference deliberations. That revision was then circulated among some 50 colleagues for opinions and advice. After three cycles of review, the resulting document is now published in this special *QSHC* supplement.^2^

## SQUIRE AND QIR

Colleagues who labour daily at improving health care in their clinical and institutional settings do not necessarily have incentives or the inclination to publish their original improvement work. Indeed, healthcare improvement is often its own reward. Academics, on the other hand, have strong incentives to publish their original work because publication provides a measure of professional productivity, one of the criteria by which they are judged by their peers and by academic institutions. Improvement researchers may use methods—for example, qualitative techniques, particularly those that reflect critical experiential learning that are less commonly used in biomedical research.^2^ SQUIRE can serve to bring these two professional worlds—healthcare improvement professionals and academics—together by offering guidance and principles for improvement experts to share their work with readers through the use of a predictable, scholarly reporting format.

SQUIRE was preceded by concise guidelines for quality improvement reports (QIR), which provided guidance for reporting local improvement projects.^4^ The QIR format supported more than 50 such reports that have been published in the scholarly literature. QIR are reports of institutional success or failure in implementation of improvement or safety strategies with reflection on the factors associated with success or failure.

Authors and editors will find that SQUIRE and QIR both emphasise the central importance of context and of the effective communication of improvement strategies.^3^^–^5 In at least two other ways, however, they serve somewhat different purposes.^6^ First, QIR offer guidance for reporting specific case examples of improvement projects, while SQUIRE is useful for reporting more extensive research into the effectiveness of improvement interventions. And, second, SQUIRE argues for employing the IMRaD (Introduction, Methods, Results and Discussion) format of scientific reports, while QIR offer flexibility for description of implementation of change. Neither SQUIRE nor QIR is an easy fit for certain kinds of healthcare improvement studies—for example, many qualitative studies, critical reviews and individual case reports.

## ROAD TESTING OF SQUIRE BY EARLY ADOPTERS

After the initial publication of the draft SQUIRE guidelines in 2005, their theory and utility underwent considerable scrutiny. The original draft guidelines were accessed online via the *QSHC* website by over 15 000 unique viewers. Authors who were early adopters^7^^–^9 have provided valuable road testing of this early draft. One positive outcome may have been that the guidelines made the underlying theory of healthcare improvement more accessible to both authors and their readers. On the other hand, a less salutary observation was that their use resulted in somewhat longer reports, which implies that the careful application of this earlier draft checklist may have burdened some authors with excessive detail in their diligent pursuit of each nuance of epistemology. SQUIRE’s initial commitment to detail and inclusiveness may have made it difficult for authors to discern the relative importance or weighting of its individual items.

Now that the revised SQUIRE guidelines are available, what are potential unforeseen and unintended consequences of SQUIRE’s wider application to healthcare improvement reports? An example of the more worrying questions include: is there a risk that improvement scholars—in their commitment to the unique epistemology of improvement science—have created potentially controversial theory that could contribute to possible isolation of this scholarly field? Might initiatives to consider deeper underlying issues of epistemology discourage valuable reporting by clinicians, administrators and scholars for whom improvement is not a first or primary discipline? The authors of SQUIRE hope it will offer guidance that, in fact, serves to circumvent these concerns and contribute to the development of a lively community of scholarly inquiry and practice about the improvement of healthcare.^2^

Nevertheless, these questions highlight the importance and value of the “Explanation and Elaboration” (E & E) document that accompanies the publication of SQUIRE in this supplement.^10^ The document was crafted by Ogrinc and colleagues to provide illustrative examples of SQUIRE components for prospective authors from the existing literature.

In addition, a SQUIRE website has been established for its promulgation and systematic evaluation.^11^ It offers opportunities for active discussion and description of the use of SQUIRE by both authors and editors.

## AN INVITATION TO USE PUBLICATION GUIDELINES MORE WIDELY IN SCHOLARLY IMPROVEMENT REPORTS

Authors and editors can readily test the utility of the SQUIRE guidelines for making healthcare improvement research more accessible to a wider audience, and the opportunities they provide for increasing the clarity of improvement research reports^1^—for example, through systematic tracking of publications that have employed SQUIRE during the process of writing and editing. The most valuable outcome would be a widening of the scholarly community for improvement—also readily measurable. Finally, we should test appropriate modifications of both SQUIRE and QIR in other reporting formats such as scientific abstracts and posters.

We encourage authors to consider the use of either SQUIRE or QIR—based on the nature of their healthcare improvement activity—as they craft their reports for *QSHC* and other scholarly journals. Finally, we encourage active interaction, specifically on the SQUIRE website and directly with the editors of *QSHC*, as the healthcare improvement community endeavours to enhance the rigour and utility of the scholarly healthcare improvement literature.

## References

[1] Stevens DP How do we know? Qual Saf Health Care 2008;17:154–5 18519618 10.1136/qshc.2008.028431

[2] Davidoff F Batalden P Stevens D Publication guidelines for quality improvement in health care: evolution of the SQUIRE project. Qual Saf Health 2008;17 Suppl 1:i3–i9 10.1136/qshc.2008.029066 PMC2773518 18836063

[3] Davidoff F Batalden F Toward stronger evidence on quality improvement. Draft publication guidelines: the beginning of a consensus project. Qual Saf Health Care 2005;14:319–25 16195563 10.1136/qshc.2005.014787 PMC1744070

[4] Moss F Thomson R A new structure for quality improvement reports. Qual Health Care 1999;8:76 10557680 10.1136/qshc.8.2.76 PMC2483636

[5] Batalden P Davidoff F What is “quality improvement” and how can it transform healthcare? Qual Saf Health Care 2007;16:1 10.1136/qshc.2006.022046 PMC2464920 17301192

[6] Thomson RG Moss FM QIR and SQUIRE: continuum of reporting guidelines for scholarly reports in healthcare improvement. Qual Saf Health Care 2008;17 Suppl 1:i10–i12 18836061 10.1136/qshc.2008.029074 PMC2602745

[7] Mooney SE Ogrinc G Steadman W Improving emergency caesarean delivery response times at a rural community hospital. Qual Saf Health Care 2007;16:60–6 17301207 10.1136/qshc.2006.019976 PMC2464913

[8] Kirsh S Watts S Pascuzzi K Shared medical appointments based on the chronic care model: a quality improvement project to address the challenges of patients with diabetes with high cardiovascular risk. Qual Saf Health Care 2007;16:349–53 17913775 10.1136/qshc.2006.019158 PMC2464960

[9] Bechtold ML Scott S Nelson K Educational quality improvement report: outcomes from a revised morbidity and mortality format that emphasised patient safety. Qual Saf Health Care 2007;16:422–7 18055885 10.1136/qshc.2006.021139 PMC2653175

[10] Ogrinc G Mooney SE Estrada C The SQUIRE (Standards for QUality Improvement Reporting Excellence) guidelines for quality improvement reporting: explanation and elaboration. Qual Saf Health Care 2008;17 Suppl 1:i13–i32 18836062 10.1136/qshc.2008.029058 PMC2602740

[11] http://www.squire-statement.org

